# Aggressive osteoblastoma of the frontal bone invading the dura: A case report and a review of related literature

**DOI:** 10.1097/MD.0000000000044076

**Published:** 2025-08-22

**Authors:** Lihao Lin, Wenhui Zhang, Yongxue Li, Yan Wang, Haoyu Shen, Yi Guan

**Affiliations:** a Department of Neurosurgery, First Hospital of Jilin University, Changchun, China.

**Keywords:** aggressive osteoblastoma, diagnosis, epithelioid osteoblastoma, skull, treatment

## Abstract

**Rationale:**

Aggressive osteoblastoma (AO) is an extremely rare cancer that mainly affects the spine and long bones and is less frequent in craniofacial bones. Cases involving the skull are even rarer, and the low incidence rates limit our understanding of the distribution and treatment strategies of AO. The aim of this study was to explore the clinical features, imaging findings, pathological characteristics, diagnoses, treatment, and prognosis of AO involving the skull.

**Patient concerns:**

A 13-year-old male was admitted to the hospital with a painless mass on the forehead that had gradually enlarged over the past year.

**Diagnoses:**

Magnetic resonance imaging of the head revealed an abnormal signal corresponding to a region with an unclear boundary measuring 2.32 cm × 2.23 cm × 2.64 cm on the left side of the frontal bone.

**Interventions:**

The entire mass was removed, and postoperative pathological examination led to a diagnosis of epithelioid osteoblastoma.

**Outcomes:**

The patient was periodically followed up for a year, and no tumor recurrence or metastasis was observed.

**Lessons:**

AO can be diagnosed on the basis of histological examination, immunohistochemical analysis, and imaging. Gross tumor resection and regular re-examination are suitable treatment options.

## 1. Introduction

Osteoblastomas originate from vascular-rich connective tissue and are characterized by the production of osteoids and woven bone surrounded by numerous osteoblasts. They are mostly benign tumors that mainly occur in the spine and long bones and rarely involve the craniofacial skeleton. The most common symptoms of osteoblastomas are swelling and localized pain, although some cases are asymptomatic and are only discovered during unrelated examinations. Osteoblastomas are currently classified into 2 clinicopathological types: traditional and aggressive osteoblastoma (AO). The former is characterized by slow growth, small lesions, and well-defined and well-vascularized lesions. In contrast, AO is more malignant and invasive than its benign counterpart and may develop from osteoid osteoblastoma (OO) or benign osteoblastoma. Furthermore, owing to the presence of atypical giant epithelioid osteoblasts and mitotic figures, AO is also called epithelioid osteoblastoma (EO), an intermediate malignant tumor between benign osteoblastoma and osteosarcoma. However, it is difficult to distinguish AO from osteosarcoma owing to its atypical histological features.^[1,2]^ Osteoblastoma accounts for about 1% of all primary bone tumors, and AO comprises only 0.28% of these tumors. AO involving the skull is rare.^[3,4]^ Due to its very low incidence, the clinical features, distribution and prognosis of AO remain unclear. Here, we report a case of AO of the frontal bone involving the dura mater and the results of a systematic review of all reported cases of AO.

## 2. Case presentation

### 2.1. History

A 13-year-old male patient presented with a painless mass on his forehead that had first appeared 1 year before and had gradually increased over that period. The mass was well demarcated with firm bone and intact skin. The patient had no reported family history of similar symptoms or related disorders. During this period, no medical intervention or treatment was administered.

### 2.2. Examination

**Figure 1.**
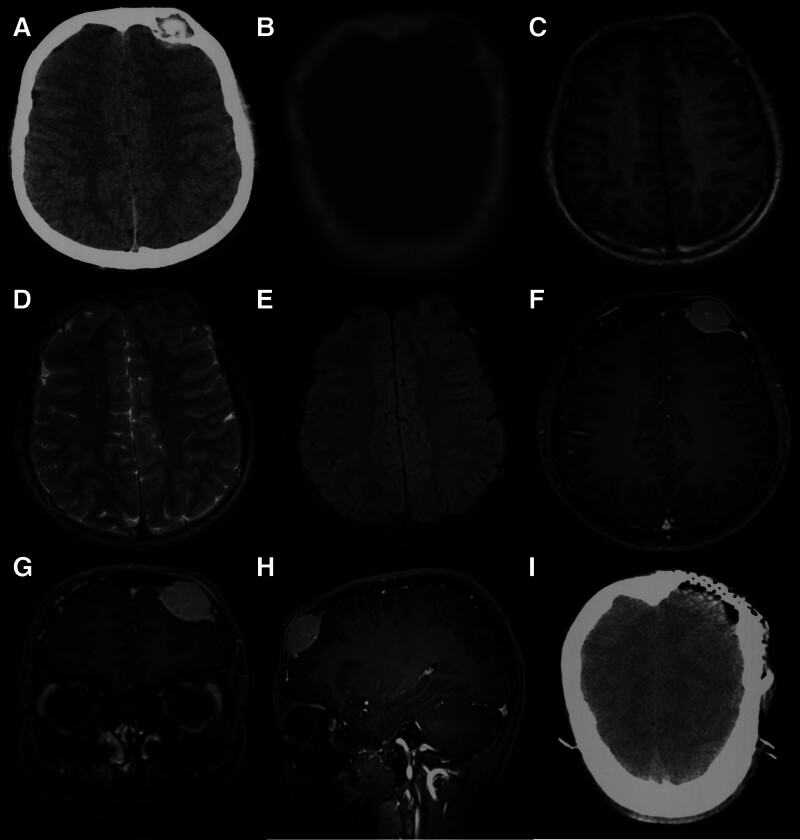
Preoperative images of computed tomography (CT) scans, magnetic resonance imaging (MRI) and postoperative images of CT. (A) CT scan of the head showing a round-shaped high and low-density shadow in the left side of the frontal bone. (B) CT bone window showing the mass penetrating the skull and invading the intracranial. (C–E) The signal is slightly lower and higher on T1WI, T2WI, and DWI. (F–H) Post-contrast MRI showing a markedly enhanced mass in the left frontal bone, along with enhancement of the adjacent meninges. (I) Postoperative CT image showing complete removal of the lesion.

Computed tomography (CT) scan of the head revealed a round high- and low-density shadow measuring 2.3 cm × 2.5 cm on the left side of the frontal bone, which protruded into the adjacent subcutaneous soft tissue and intracranial space (Fig. 1A and B). Furthermore, magnetic resonance imaging (MRI) of the head indicated an abnormal signal measuring 2.32 cm × 2.23 cm × 2.64 cm on the left side of the frontal bone. The boundary was unclear and the signal was uneven hypointense-to-hyperintense on T1WI, T2WI, and DWI (Fig. 1C–E). After gadolinium administration, obvious heterogeneous enhancement was observed and the adjacent dura was also enhanced (Fig. 1F–H). No abnormal signals or enhancement shadows were observed in the remaining brain tissues. Routine examination after admission showed an alkaline phosphatase (ALP) level of 221 U/L, which was significantly higher than the normal range of 45 to 125 U/L. The other tests did not reveal any obvious abnormalities.

### 2.3. Surgery and pathological finding

Based on imaging findings, the initial diagnosis was a common osteoma. A decision was made to perform frontal bone mass resection based on the imaging findings. The tumor was completely removed, and titanium mesh cranioplasty was performed to repair the frontal bone defect (Fig. 1I). A subcutaneous drainage tube was placed, and the incision was sutured. Postoperative wound healing was good with no obvious discomfort.

**Figure 2.**
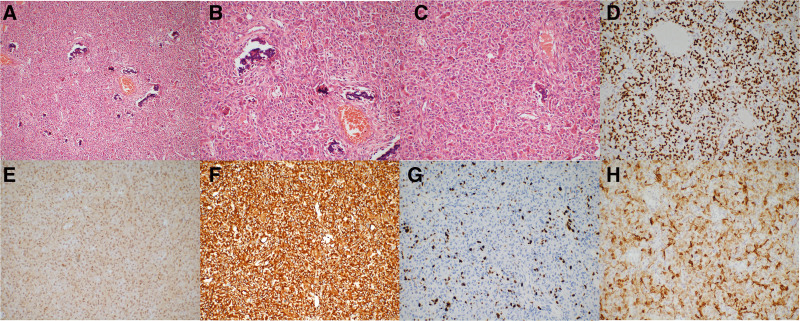
Histopathological examination of the excised tumor. (A–C) Light microscopy images showing a relatively uniform number of epithelioid osteoblasts with eosinophilic cytoplasm, nuclear deviation, and prominent nucleoli, (A) ×10, (B) ×20, (C) ×20. (D–H) Immunohistochemical staining for (D) SATB2, (E) MDM2, (F) Ki-67, (G) vimentin, and (H) desmin.

A pathological report showed an EO of the skull. The tumor volume was 3 cm × 2.8 cm × 1.2 cm. No tumor was found in the skull margin, and meningeal tissue sent for examination showed tumor involvement (Fig. 2A–C). The cells showed eosinophilic cytoplasm, nuclear deviation, and prominent nucleoli. Mitosis was rare and reactive osteogenesis and microvascular hyperplasia were observed. Immunohistochemical staining showed that the mass was positive for Ki67 (10%), vimentin, CD68, desmin, CD30, SATB2, SSTR2, CD31, and CD34 and negative for MDM2, CDK4, S100, EMA, SMA, CD1a, CD163, H3.3G34W, H3K36M, My0D1, myogenin, langerin, P63, CKpan, PR, ALK, GFAP, and LCA. SATB2 is a marker of osteoblast differentiation, and the absence of MDM2 differentiates AO from benign bone tumors with bone formation (Fig. 2D, E). Furthermore, 10% Ki67^+^ cells indicated that the tumor was more aggressive than traditional osteoblastoma (Fig. 2F). Overexpression of vimentin and desmin in tumors was closely related to tumor growth, invasion, and poor prognosis (Fig. 2G, H).

### 2.4. Postoperative course

On the basis of these findings, no special treatment was prescribed by the Department of Radiotherapy and Pediatric Oncology. The patient was regularly reviewed after discharge, and no tumor recurrence or metastasis was detected during the 1-year follow-up.

## 3. Discussion

### 3.1. Background

Aggressive osteoblastomas were first observed by Dorfman^[5]^ and subsequently described as malignant osteoblastomas by Schajowicz and Lemos.^[6]^ Dorfman and Weiss reviewed 15 cases of AO in 1984 and defined these lesions as borderline tumor entities that are distinct from low-grade osteosarcoma and have high recurrence rates and malignant potential.^[7]^ Due to its low incidence rate, research on AO is limited. Therefore, we conducted a systematic literature review of reported cases of AO in the PubMed database. Relevant studies were searched using the keywords “aggressive osteoblastoma” and “epithelioid osteoblastoma,” and 196 case reports were retrieved (Table S1, Supplemental Digital Content, https://links.lww.com/MD/P749).

### 3.2. Epidemiology

The clinical features of the patients are summarized in Table 1. After excluding cases of unknown sex, we found that 62.7% of AO cases occurred in males, whereas 37.3% of the patients were female. The youngest recorded male AO patient was 2 years old,^[8]^ and the oldest was 80 years old,^[7]^ with an average age of 26 years. Among female patients, the youngest was 5 years old and the oldest was 72 years old,^[9]^ with an average age of 27.4 years. Furthermore, male patients were generally younger than female patients at the time of the disease onset. The most common site of AO was the trunk bone, with 69 recorded cases (35.2%), including 5 cases where the ribs were affected, 1 case of sternum, and 63 cases of spinal lesions. The cervical spine was affected in 20 patients, cervicothoracic spine in 2, thoracic spine in 19, lumbar spine in 10, and sacral spine in 8. The lower limb bone was affected in 52 cases (26.5%), including the hip in 15, femur in 14, tibia in 8, fibula in 4, and feet in 11. There were 16 cases (8.2%) of upper limb bones and 33 cases (16.8%) of maxillofacial bones, including 14 cases of the maxilla and 19 cases of the mandible. Only 18 cases (9.2%) of AO in the skull have been reported so far, of which the frontal bone, temporal bone, sphenoid bone, and parietal and occipital bones were affected in 4, 6, 1, and 4 cases respectively (Table 2). In rare cases, lesions have also been observed in the breast, thyroid cartilage, and sinuses. Approximately 70% of traditional osteoblastoma cases involve only one vertebral body, whereas 50% of AO cases involve both.^[4]^ The smallest diameter recorded for an AO lesion was 0.5 cm,^[23]^ and the largest reported tumor measured 20 × 7 × 7.2 cm and was detected in the middle of the tibia.^[24]^ Della Rocca et al examined 55 cases of osteoblastoma and concluded that the clinical aggressiveness of the tumors is related to the bone location rather than specific histological features.^[25]^

**Table 1:** Clinical features of all 196 aggressive osteoblastoma (AO) patients.

Location	Cases	Percent (%)	Sex		Mean age (years)	Mean size (cm)	Mean duration of symptoms (m)	Treatment			Recurrence	Metastasis
			Male	Female				GTR	STR	N/A		
Skull	18	9.2	12	6	29.0	5.29	12.2	11	4	3	6	0
Maxilla	14	7.1	8	5	14.7	4.21	7.5	6	2	6	2	0
Mandible	19	9.7	12	7	23.3	4.23	13.0	9	4	6	1	0
Sinuses	2	1.0	1	1	16.5	N/A	19.3	1	1	0	1	0
Rib	5	2.6	2	3	30.8	5.38	N/A	3	N/A	2	0	0
Sternum	1	0.5	1	0	27.0	9.2	9.0	N/A	N/A	1	N/A	N/A
Cervical	20	10.2	15	5	23.8	3.6	9.0	9	10	1	2	0
Cervicothoracic spine	2	1.0	2	0	45.5	N/A	5.0	2	0	0	1	0
Thoracic	19	9.7	12	7	25.7	3.54	N/A	4	11	4	4	0
Lumbar	10	5.1	8	2	23.3	4.36	3.0	4	6	0	2	0
Sacrum	8	4.1	6	2	25.4	5.5	16.3	2	3	3	2	0
Unknown vertebral body	4	2.0	2	2	26.5	N/A	N/A	N/A	N/A	1	N/A	0
Upper limb bone	16	8.2	10	5	26.8	4.94	8.8	7	2	7	3	1
Lower limb bone	52	26.5	27	25	26.8	6.1	11.7	20	11	21	11	1
Throat	2	1.0	1	N/A	48.5	3.0	10	1	N/A	1	0	0
Breast	1	0.5	0	1	65.0	2.5	N/A	1	0	0	0	0
Skin and soft tissue	3	1.5	2	1	30.3	2.47	12	3	0	0	1	0

GTR = gross total resection, N/A = not available, STR = subtotal resection.

**Table 2:** Aggressive osteoblastoma in the skull.

Authors	Age	Sex	Location	Symptoms	Size (cm)	Calcification in imaging	Treatment	Recurrence	Metastasis	Follow-up (months)
	(years)									
Pitlyk and Guichard^[10]^	26	M	Left frontal lobe	Pain for 8 weeks	10 × 12.5 × 2	N/A	GTR	NO	NO	7/ANED
Dorfman and Weiss^[7]^	60	F	Ethmoid bone	N/A	N/A	N/A	N/A	N/A	N/A	N/A
Dorfman and Weiss^[7]^	26	M	Skull	N/A	N/A	N/A	N/A	N/A	N/A	N/A
Adler et al^[11]^	28	F	Right temporal bone/base of middle cranial fossa	Pain swelling, cramping, facial paralysis 11 months	N/A	N/A	GTR	6 months	NO	64/AWD
Lin et al^[12]^	55	F	Right frontal and parietal bones	Swelling 7 months	Parietal bone 2.6 × 3.2 × 2.7	N/A	GTR	NO	NO	6/ANED
					Frontal bone 1.5 × 1.0 × 0.5					
Kukwa et al^[13]^	12	F	Sphenoid bone	Protrusion of the eyes, headache for 2 months	5.5 × 3.5 × 5.5	Yes	GTR	3 months	N/A	61/ANED
Lu et al^[14]^	18	M	Left temporal bone	Pain and swelling for 12 months	3.3 × 3.0	Yes	GTR	NO	NO	12/ANED
Nord et al^[15]^	35	M	Skull	N/A	N/A	N/A	N/A	21 months	NO	60/ANED
Jain et al^[16]^	32	F	Left temporal bone	Swelling for 6 months, hearing loss, left-sided facial paralysis grade II, unsteady walking	5.0 × 5.0	N/A	GTR	N/A	N/A	N/A
Singh et al^[3]^	34	M	Foramen magnum to axis	Painful swelling for 5 months, voice changes, dysphagia, and left upper extremity weakness within 3 months	5.3 × 6.2 × 5.5	Yes	STR	NO	N/A	5/ANED
Mohanty et al^[17]^	23	M	Temporal bone	Swelling and pain	3.0 × 2.0	N/A	GTR	6 months	N/A	18/ANED
Kraft et al^[18]^	49	M	Right temporal bone	Right hearing loss for 18 months, facial and forehead pain for 2.5 months	N/A	N/A	STR	4 months	N/A	24/DOD
Dixit et al^[19]^	20	M	Left temporal bone	Hearing loss, ringing in the ears, painful swelling, right mouth deviation for 4 years	5.0 × 6.0 × 5.5	N/A	STR	N/A	N/A	N/A
Jacques et al^[20]^	50	M	Left frontal parietal bone	Swelling with headache	Intracranial 3.9 × 6.7 × 4.6	Yes	GTR	NO	NO	60/ANED
					Extracranial 4.7 × 2.4					
Toescu et al^[21]^	20	F	Right frontal bone	1 year of swelling, recent vomiting	7.5 × 6.0	N/A	GTR	NO	NO	24/ANED
Attiah et al^[2]^	18	M	Left middle cranial fossa	Left temple mass with left ear filling for 3 weeks	5.9 × 4.2 × 5.8	N/A	STR	NO	NO	27/ANED
Sharma et al^[22]^	18	M	Right parietal occipital region	Swelling	9.0 × 8.1 × 8.0	N/A	GTR	15 months	NO	21/AWD
Present case	13	M	Frontal bone	Swelling 12 months	2.32 × 2.23 × 2.64	N/A	GTR	N/A	N/A	12/ANED

ANED = alive without evidence of disease, AWD = alive with disease, DOD = dead of disease, F = female, GTR = gross total resection, M = male, N/A = not available, STR = subtotal resection.

Although the exact etiology of AO is unknown, viral infections, vascular abnormalities, and physical trauma have been implicated. Among the reviewed cases of AO, 5 occurred after trauma or surgical operation, and the lesion site had a long history of injury and chronic progression.^[10,20,21,23,26]^ In addition, there were 4 cases with secondary aneurysmal bone cysts.^[20,27–29]^

### 3.3. Clinical presentation

The earliest symptoms include local pain, swelling, radiating pain, muscle spasms, and limb numbness. Pain is generally not aggravated at night, and most patients are not sensitive to nonsteroidal anti-inflammatory drugs such as aspirin. However, in a study conducted on 19 AO patients, Jiang et al reported that 4 of the 9 patients (44%) who received nonsteroidal anti-inflammatory drugs experienced partial pain relief.^[30]^ Furthermore, most AO cases involving the skull can induce neurological symptoms such as facial paralysis, hearing loss, and limb muscle weakness.^[3,16,19]^ In cases with spinal involvement, neurological symptoms corresponding to the spinal plane may also occur in cases of spinal involvement.^[9,31,32]^ We found that the average duration of overall symptoms in patients with AO was about 11 months. While pain alone can last for an average of 20.7 months in patients with traditional osteoblastoma, the average duration of pain in patients is only 10.8 months. Patients with AO also presented with symptoms nearly a year earlier than those with traditional osteoblastoma, generally experienced more blunt pain, and had a 31.7% higher risk of developing neurological dysfunction.^[4,33]^ AO can cause significant bone destruction, soft tissue infiltration and epidural invasion, which is challenging to operate. AO of the skull typically presents with progressive localized swelling, usually originating in the medulla, which is largely painless at the beginning and becomes painful as the lesion grows and reaches the periosteum.

### 3.4. Imaging findings

Bone tumors can be divided into 3 stages according to imaging and clinical manifestations. Most osteoblastomas are Enneking stage 2 lesions, whereas AO is classified as an Enneking stage 3 tumor characterized by active and regionally aggressive lesions with extracapsular or extramedullary extension.^[34]^

AO may appear expandible on CT examination, with stromal calcification and cortical bone destruction. Radiologically, AO is similar to aneurysmal bone cysts and osteosarcomas.^[35,36]^ Currently, CT is the gold standard for the diagnosis and treatment of AO.^[37]^ MRI can also show the medulla, soft tissue, cystic degeneration, hemorrhage, and the extent of the tumor. Owing to the large amount of loose fibrous connective tissue and abundant vascular stroma, most AOs are hypo- to iso-dense on unenhanced images, slightly hypo-to-isointense on T1WI, and hyperintense on T2WI. After gadolinium administration, obvious heterogeneous enhancement was observed, and ossification and calcification were common in AO lesions. In our review, we found 36 cases (18.4%) with radiographic description of this phenomenon, of which 4 cases (22.2%) involved the skull and showed higher ossification and calcification compared to the lesions at other sites (Table 2). However, the grade of bone tumors diagnosed using MRI was higher than that diagnosed using CT. Huang et al examined 21 osteoblastoma lesions by CT and diagnosed 6 cases as Enneking stage 3 tumors. Furthermore, 19 of these cases were simultaneously examined using MRI, and 18 were diagnosed with stage 3 disease. Thus, CT and MRI findings were consistent in only 31.6% of cases. Nevertheless, AO was confirmed in 5 cases on the basis of postoperative pathological examination, indicating that CT results are more consistent with the actual diagnosis.^[38]^ Although AO of the skull is radiographically similar to other traditional osteoblastomas, intracranial progression in the former can destroy the cerebral cortex, resulting in seizures, increased intracranial pressure, nausea, vomiting, and headaches.^[21]^

Compared to MRI and CT, bone scintigraphy may be a more accurate diagnostic approach for AO,^[33]^ although the final diagnosis requires postoperative histopathological confirmation.

### 3.5. Pathological and genetic characteristics

AO masses can invade adjacent soft tissues and form prominent, irregular trabeculae and disordered osteoid matrices.^[39]^ In a review of histological findings, Oliveira et al confirmed the presence of AO as a separate entity from traditional osteoblastoma and found that chain or sheet osteoid deposits were associated with the recurrence and aggressive behavior of AO.^[40]^ Nord et al reported that osteoblastoma lesions have little or no genomic changes, as opposed to the significant genomic rearrangements in AO,^[15]^ especially balanced translocations on chromosomes 4, 7, and 14.^[41]^ In the report by Pereira et al, all cases were positive for FOS immunoreactivity; however, FOSB immunostaining showed nuclear positivity only in a few scattered epithelioid osteoblasts.^[42,43]^ Among the 15 patients with the relevant description, 83% were FOS-positive, 67% were FOSB-positive, and 56% were positive for both. Panagopoulos et al detected a COL1A1-FYN fusion gene in EO cells, resulting in dysregulated FYN expression. Thus, the COL1A1-FYN fusion may represent a novel genetic signature of AO.^[44]^

### 3.6. Examination

Increased ALP level is an essential feature of AO. Yin et al reported supranormal (normal value 45–125 U/L) preoperative ALP levels in AO patients, which decreased significantly after surgical resection of the lesion.^[4]^ The preoperative ALP level in our patient was 221 U/L, which dropped to 169.5 U/L after tumor removal. Furthermore, 4 cases in the reviewed studies had elevated ALP levels before surgery.^[21,45–47]^ Morris reported a case of AO with high preoperative beta-human chorionic gonadotropin (β-hCG) levels. After excluding pregnancies, a postoperative review reported a decrease in β-hCG levels. In addition, the presence of β-hCG in these tumors and sarcomas is associated with poor prognosis.^[48]^

### 3.7. Differential diagnosis

The definitive diagnosis of AO also depends on its differentiation from that of OO. In our review, we found that 3 cases of OO transformed to AO at a later stage,^[49–51]^ and the incidence of OO was 4 times higher than that of osteoblastomas.^[52]^ OO can usually be detected and treated early in the disease and forms smaller lesions than AO. This can be attributed to the fact that OO mainly involves the cortex of the bone and is close to the richly innervated periosteum, which produces the corresponding sensory stimulation that helps in early tumor detection. Furthermore, the dense cortex limits rapid growth of the tumor, resulting in smaller lesions. However, AO is more likely to involve the medulla and lacks cortical bondage and sensory innervation, which not only increases the size of the lesions but also delays their detection.^[53]^ The local recurrence rate of OO is 5% to 10% compared to 27.3% observed with AO. Although epithelioid osteoblasts are found in both OO and traditional osteoblastomas, they do not appear in the trabecular spaces as they do in AO.

Osteoblastoma-like osteosarcoma and AO are highly invasive and malignant, making the differential diagnosis challenging. AO is characterized by EO cells surrounding osteoid trabecular bone, and the presence of solid sheets filling the trabecular space in some cases. Osteoblastoma-like osteosarcoma exhibits an invasive growth pattern wherein tumor cells infiltrate into the original lamellar bone, forming a mass with unclear boundaries and resulting in cortical destruction.^[48]^ In fact, osteoblastoma-like osteosarcoma masses have more obvious ill-defined borders on imaging.^[54]^

### 3.8. Treatment modalities

Owing to the abundant blood supply in AO, complete resection may be difficult in some patients. Schur et al reported a case of AO wherein preoperative embolization of the main supplying vessel followed by complete tumor resection led to a significant reduction in blood loss compared with similar previous procedures.^[55]^ Radiofrequency ablation (RFA) is an effective palliative treatment for osteoblastomas with low complication rates and mild symptoms.^[56,57]^ However, the efficacy of RFA remains to be confirmed. Reynolds et al treated one case of AO with RFA and performed surgical resection later as the tumor grew larger than before.^[32]^ Cryoablation has several advantages over RFA, such as real-time monitoring of the treatment area that avoids the destruction of normal tissues, less pain, the possibility of using multiple probes simultaneously, and greater efficiency owing to the creation of an ice ball that fits the size and shape of the tumor.^[37]^ Moreover, carbon-ion radiation therapy can trigger complex DNA double-strand breaks and is therefore a promising treatment option for AO.^[58]^

Several new therapeutic strategies for AO are currently under development. For instance, Kooner et al showed that denosumab inhibits osteoclast-mediated bone destruction, which shifts the balance between the bone-forming stage and the transformation of malignant masses into solid sclerosing lesions.^[59]^ Zhu et al further demonstrated that endoplasmic reticulum stress contributes to bufotalin-induced osteoblastoma cell death in animal models, indicating that bufotalin may also have therapeutic effects against AO.^[60]^ There have also been reported to inhibit the activity of osteoclasts, reduce the proliferation of tumor cells, and induce apoptosis.^[61]^ Camitta et al described a case of postoperative recurrence of osteoblastoma, wherein high-dose methotrexate, doxorubicin, and cisplatin rapidly inhibited tumor growth, and no tumor recurrence was observed within 33 months of follow-up after cessation of chemotherapy.^[62]^ Thus, although there is currently no consensus on chemotherapy, it may be helpful in some patients with recurrent aggressive tumors or in those who cannot be treated surgically.

### 3.9. Prognosis

A literature review indicated that the recurrence rate of AO was 26.7%, and the mean time to recurrence was 12.4 months after surgery. The recurrence rate of skull lesions was higher (42.9%), and the average recurrence time was 9.2 months, thus making it more malignant than tumors affecting other sites (Table 2). Following gross total resection, the recurrence rate was 23%, and the mean recurrence time was 14.4 months. However, subtotal resection results in a 34.1% recurrence rate and a mean recurrence time of 8.1 months. Thus, complete resection of the tumor mass is crucial to avoid postsurgical recurrence of AO. The longest relapse-free time was 444 months in all cases.^[63]^ Despite its high recurrence rate, only 2 cases of metastasis have been reported so far, and the lungs were the affected site in both cases.^[64,65]^ In addition, there has been one reported case of AO transforming into osteosarcoma.^[64]^

Therefore, patients with AO should undergo en-bloc resection to ensure the removal of all affected soft tissues. If the anatomical location of the tumor is not suitable for complete resection, subtotal resection should be performed first, followed by RT. Studies have shown that the long-term recurrence-free survival after radiotherapy is as long as 25 years.^[66]^ Radiotherapy either stops tumor growth or partially shrinks the mass to reduce clinical effects. However, there is still no consensus on whether radiotherapy can reduce the recurrence rate of AO, and some researchers believe that adjuvant radiotherapy may cause osteonecrosis or even lead to the transformation of residual tumor into more malignant osteosarcoma.^[2]^ Nevertheless, Attiah et al were able to treat residual tumors in the temporal bone with adjuvant proton radiation therapy, and no tumor progression was observed during a follow-up period of >2 years.^[2]^ In addition, there is evidence that higher preoperative ALP levels correlate with larger tumors and a higher probability of recurrence.^[4]^

Patients with AO require more frequent and long-term clinical and radiological follow-ups. Among all recurrence cases, 92% occurred within the first 2 years of treatment. Therefore, we recommend that patients undergo follow-up evaluations either quarterly or semiannually during the initial 2 years, followed by annual assessments thereafter. Even in patients who have undergone total resection, prompt review should be performed to detect recurrence at an early stage and provide timely treatment.

## 4. Conclusions

In summary, only a few cases of AO have been reported in the literature, and cases occurring in the skull are even rarer. Thus, more cases describing OA, as well as long-term follow-up studies, are needed to fully understand AO. Therefore, because of these limitations, the present case report might represent an additional reference among the few available that might serve as a potential guide for clinicians and radiologists.

## Author contributions

**Conceptualization:** Lihao Lin, Yi Guan.

**Data curation:** Yongxue Li.

**Formal analysis:** Yan Wang, Yi Guan.

**Funding acquisition:** Yi Guan.

**Investigation:** Wenhui Zhang, Yan Wang.

**Methodology:** Haoyu Shen.

**Resources:** Yi Guan.

**Software:** Yongxue Li.

**Supervision:** Yi Guan.

**Validation:** Yi Guan.

**Visualization:** Lihao Lin.

**Writing – original draft:** Lihao Lin, Wenhui Zhang, Yi Guan.

**Writing – review & editing:** Lihao Lin, Yi Guan.

## Supplementary Material


